# Australian Notes

**Published:** 1919-04-26

**Authors:** 


### AUSTRALIAN NOTES.

### About Nurses.

Hospitals continued to send replies to the State Treasurer as to the proposed eight-hours' day for nurses, in the first part of October. All declared that it was simply a matter of money and accommodation. Since then the matter has retired into the background.

Nurse Ettie Richards returned to Melbourne lately on six months' furlough. For sixteen months she has been on duty at the Deolali Military Hospital, some 150 miles from Bombay, which accommodates 5,000 patients. When first established the hospital was maintained by the people of Wales and staffed by Welsh doctors and nurses. Now a Victorian, Nurse Alma Bennet, is in charge with a staff of Australian nurses, the patients being British and Australian from Mesopotamia, and wounded Turks, prisoners of the battlefield.

References to the splendid work of the Australian nurses in India and Burma was recently cabled from Bombay to our Minister for Defence: "All had won great praise, and Principal Matron G. M. Davis—in charge of the Australian Army Nursing Service in India—has been mentioned in despatches." Formerly attached to the staff of the Caulfield Military Hospital, she left for India two years ago.

### Intermediary Hospitals.

For the last fifteen years three successive inspectors of charities have advocated the establishment of Government intermediary hospitals to relieve the pressure on eleemosynary hospitals by those who could pay a moderate fee. Such a hospital had a few months' trial in Melbourne some time ago, but supporters of the system refused to regard the test as fair or sufficient. The State Treasurer (Mr. McPherson) is now obtaining information on the working of the scheme from the Dominion of New Zealand, though, as he took care to remark, this did not mean that intermediary hospitals were to be established in Victoria at once.

### Vaccines.

The Commonwealth of Australia as a producer of vaccines—including on against Spanish influenza—is an established fact. The Federal serum laboratories are now prepared to place on the market good supplies of vaccines formerly imported from abroad, and these laboratories, it is asserted, will be capable of producing all that may be required. But at present the way is blocked. On October 22, it was made known that wholesale chemists were refusing to buy stocks for distribution, as they were dissatisfied with the discount offered by the Quarantine Service. The difference between the discount offered and that sought is understood to be from 7 to 8 per cent. The matter is now under consideration by the Minister of Customs, as head of a department controlling laboratories.

### V.D. Act.

The inquiry by Special Commissioner into the conduct and management of the Greenvale Sanatorium, Victoria, has resulted, as was anticipated, in a verdict of "not proven."

All hospitals in Melbourne are reporting crowded conditions. On October 23, 400 in-patients were receiving treatment in the Melbourne Hospital—a record since its establishment. At the mental hospitals, the State Treasurer and the Chief Secretary found unused buildings at Kew and Yarra Bend Asylums which are to be brought into use to relieve the strain.

### As One Result.

Australia and New Zealand have been designated as "the home of catarrh," owing to the changeable climate of this portion of the South Pacific. Also, it was questioned whether sufficient precautions were taken by these two countries to keep out trachoma, and the Conference decided to forward the following "recommendation" to the Australian (Federal) Government: "That strict examination be made for the presence of trachoma and other eye diseases on all passengers and crews of all vessels from overseas (not previously resident in Australia), and that no person be allowed to land if affected with disease of a contagious character; any person affected by the regulation to have the right of appeal to a qualified ophthalmic surgeon."

